# Correction: Highly contiguous assemblies of 101 drosophilid genomes

**DOI:** 10.7554/eLife.78579

**Published:** 2022-03-18

**Authors:** Bernard Y Kim, Jeremy R Wang, Danny E Miller, Olga Barmina, Emily Delaney, Ammon Thompson, Aaron A Comeault, David Peede, Emmanuel RR D'Agostino, Julianne Pelaez, Jessica M Aguilar, Dilêr Haji, Teruyuki Matsunaga, Ellie Armstrong, Molly Zych, Yoshitaka Ogawa, Marina Stamenković-Radak, Mihailo Jelić, Marija Savić Veselinović, Marija Tanasković, Pavle Erić, Jian-Jun Gao, Takehiro K Katoh, Masanori J Toda, Hideaki Watabe, Masayoshi Watada, Jeremy S Davis, Leonie C Moyle, Giulia Manoli, Enrico Bertolini, Vladimír Košťál, R Scott Hawley, Aya Takahashi, Corbin D Jones, Donald K Price, Noah Whiteman, Artyom Kopp, Daniel R Matute, Dmitri A Petrov

**Keywords:** *D. melanogaster*, Other

Kim BY, Wang JR, Miller DE, Barmina O, Delaney E, Thompson A, Comeault AA, Peede D, D'Agostino ERR, Pelaez J, Aguilar JM, Haji D, Matsunaga T, Armstrong EE, Zych M, Ogawa Y, Stamenković-Radak M, Jelić M, Veselinović MS, Tanasković M, Erić P, Gao J-J, Katoh TK, Toda MJ, Watabe H, Watada M, Davis JS, Moyle LC, Manoli G, Bertolini E, Košťál V, Hawley RS, Takahashi A, Jones CD, Price DK, Whiteman N, Kopp A, Matute DR, Petrov DA. 2021. Highly contiguous assemblies of 101 drosophilid genomes. *eLife* **10**:e66405. doi: 10.7554/eLife.66405.

Published 19 July 2021

This review was prepared by Bernard Kim, Dilêr Haji, Noah Whiteman, Artyom Kopp, Daniel Matute, and Dmitri Petrov.

This correction is issued to correct the species identification of the *Drosophila nebulosa* 14030–0761.01 line from this study. Here, we show that strain 14030–0761.01 is not *D. nebulosa*, but instead *D. sucinea*, and that the line is likely misidentified at the stock center. It is unknown whether line 14030–0761.01 was originally *D. nebulosa*. Although we did not assemble the genome of *D. sucinea* 14030–0791.00, our analyses of genomic data from this strain also show that it is likely misidentified *D. paulistorum*.

Several genomes of drosophilid species belonging to the *willistoni* species group were assembled for this study. Among these were two closely related species purchased from the National *Drosophila* Species Stock Center (NDSSC) in December 2019: *D. sucinea* 14030–0791.01 and “*D. nebulosa”* 14030–0761.01. For clarity, we will refer to misidentified strains henceforth with the species name in quotes. After the publication of this manuscript, we were notified that our assembly of “*D. nebulosa”* 14030–0761.01 resembled *D. sucinea* more than other *willistoni* group species (pers. comm. Christopher Sottolano, Anthony Geneva, and Nir Yakoby). Indeed our “*D. nebulosa”* genome appears as a sister taxon to *D. sucinea* in our phylogeny, rather than the other *willistoni* group species we sequenced (Figure 5 of the original manuscript), inconsistent with other phylogenies inferred from molecular data (e.g., Finet et al., 2021).

We first wished to eliminate the possibility that we unknowingly sequenced *D. sucinea* multiple times due to sample mishandling. If so, the variation present in the long and short read datasets should not be consistent with two genetically distinct lines. To test for this, we built new genome assemblies to obtain a consensus sequence of the variation represented by each set of reads, then inferred the phylogenetic relationships of the new assemblies.

In addition to the *willistoni* group assemblies already generated through the hybrid approach in our previous work (including *D. sucinea* and “*D. nebulosa”*), we newly assembled our Nanopore and Illumina reads for *D. sucinea* 14030–0791.01 and “*D. nebulosa”* 14030–0761.01, and Illumina reads for “*D. sucinea”* 14030–0791.00 and *D. nebulosa* 14030–0761.00 (Khallaf et al., 2021; available from NCBI BioProject PRJNA669609). However, “*D. sucinea”* 14030–0791.00 was ignored for reasons we will cover shortly. We also obtained an unpublished draft assembly of *D. nebulosa* 14030–0761.06 courtesy of Christopher Sottolano and Nir Yakoby. Finally, our *D. saltans* assembly was used as an outgroup. Short read datasets were assembled with SPAdes v3.15.3 (Prjibelski et al., 2020). Nanopore reads were assembled with Flye 2.9 (Kolmogorov et al., 2019) then polished once with Oxford Nanopore’s Medaka software (v1.4.4). The 125 BUSCO genes (Manni et al., 2021) that were the most complete across all assemblies were used to build an ASTRAL tree (Zhang et al., 2018), using the methods from our study (Figure 1).

**Figure 1.**
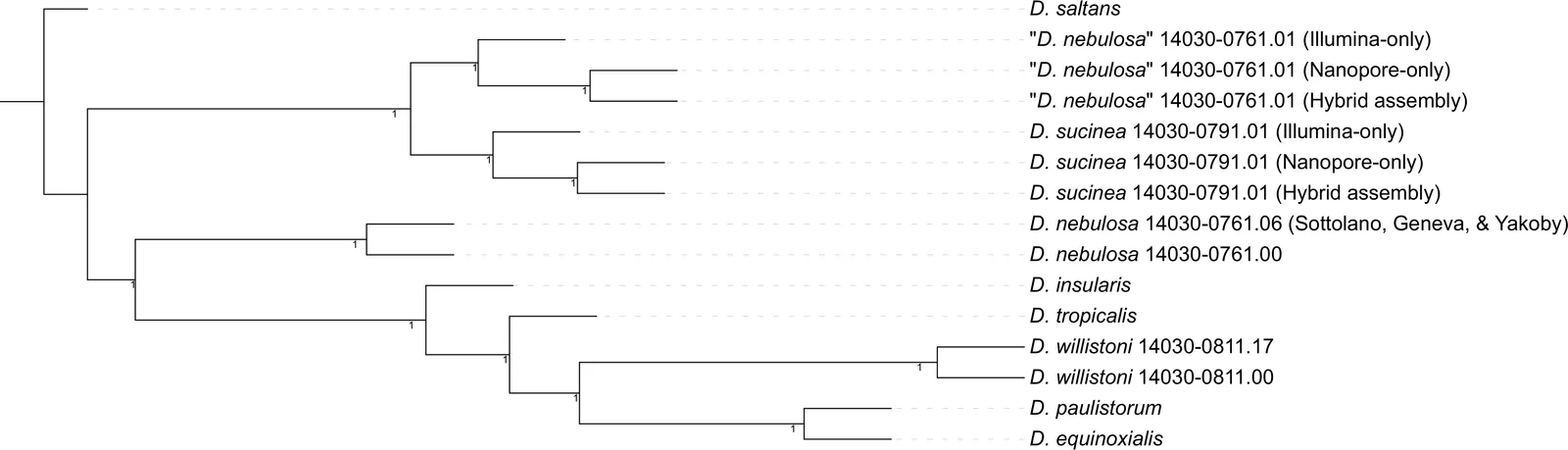
A phylogenetic tree constructed with genomic data from various *willistoni* group species suggests that line 14030–0761.01 is misidentified. The ASTRAL tree is constructed from 125 randomly selected complete single-copy BUSCOs from each tip genome. Node confidence values are the local posterior probabilities of each node.

The phylogenetic relationships between the various samples showed us two important things (Figure 1). First, “*D. nebulosa”* 14030–0761.01 is indeed more closely related to *D. sucinea* than to the other *D. nebulosa* assemblies, confirming our suspicions that this line is misidentified. Second, the sequences from “*D. nebulosa”* 14030–0761.01 and *D. sucinea* 14030–0791.01 form clusters distinct from each other, meaning the samples were properly handled for sequencing.

While we originally downloaded “*D. sucinea”* 14030–0791.00 data from NCBI for these analyses, we found those data to be also inconsistent with the phylogeny and ignored them for this analysis. COI sequences extracted from these reads and queried at the Barcode of Life Database (Ratnasingham and Hebert, 2007) suggested these reads were instead from *D. paulistorum*. Mapping these sequences against our *willistoni* group assemblies was consistent with this species prediction: only 27.9% of reads mapped to our *D. sucinea* assembly while the best-mapping assembly was *D. paulistorum* 14030–0771.06, with 97.5% of reads mapped.

Although strain misidentification seems to explain the anomaly in our data, we sought to further clarify the species identity of the strain and the origin of the misidentification or contamination. To eliminate the possibility of contamination in strains maintained in our labs, we ordered nine fresh lines from the NDSSC: (four lines) *D. nebulosa* 14030–0761.00,01,03,06; (three lines) *D. sucinea* 14030–0791.00,01,02; *D. capricorni* 14030–0721.01; and *D. willistoni* 14030–0811.17. Sanger sequencing of the COI marker locus was performed for each strain and wings were examined for an anterior dark spot (Figure 2), a distinguishing characteristic of *D. nebulosa* (pers comm. A Kopp).

**Figure 2.**
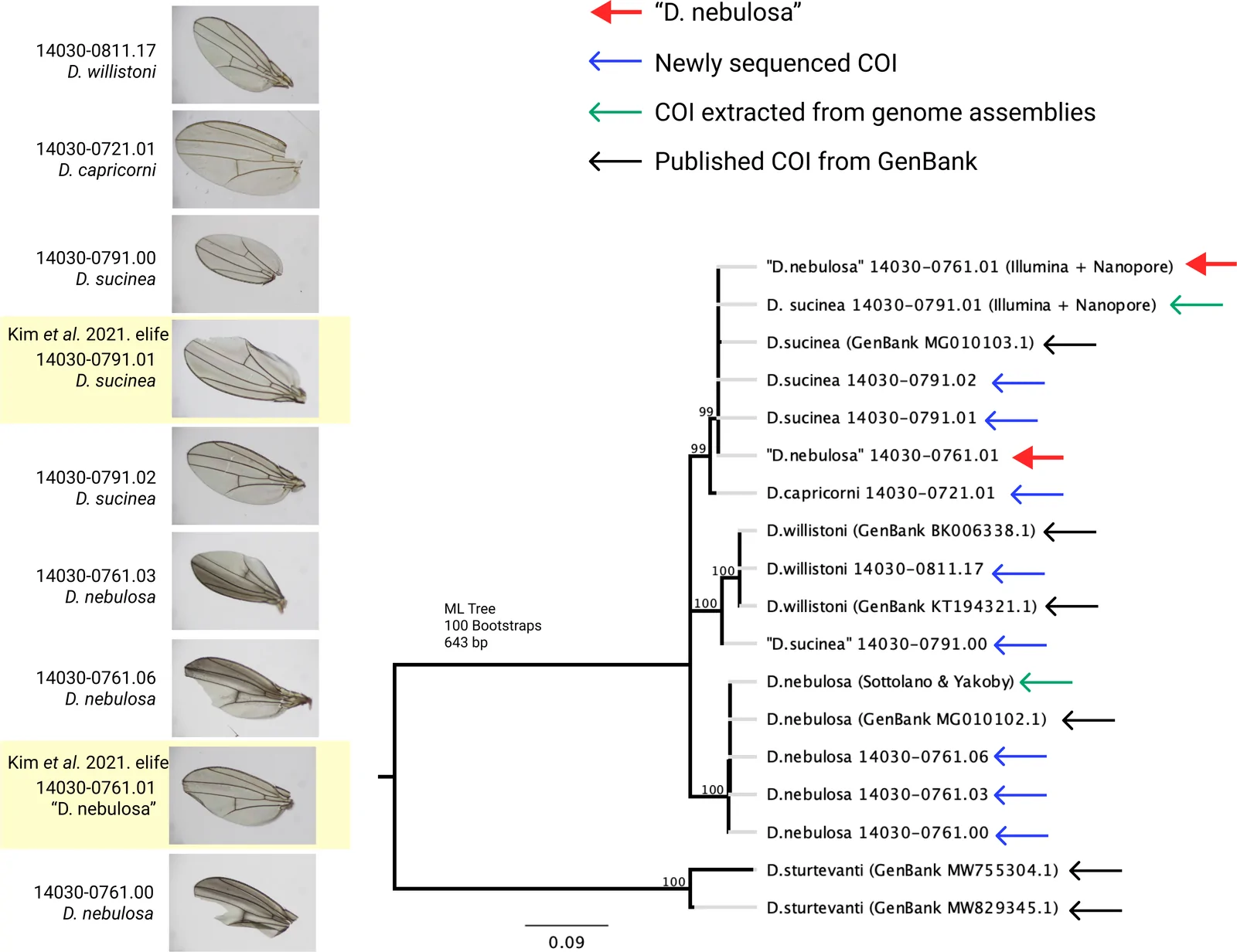
Wing pigmentation and inferred COI sequence relationships indicate species misidentifications. The wing coloration of *“D. nebulosa”* 14030–0761.01 is not consistent with the darker pigmentation observed in other *D. nebulosa* strains. Similarly, a maximum likelihood phylogeny of 18 COI sequences shows that *“D. nebulosa”* 14030–0761.01 and *“D. sucinea”* 14030–0791.00 are likely to be misidentified.

As expected, “*D. nebulosa”* 14030–0761.01 lacks the anterior pigmentation found in true *D. nebulosa* lines (Figure 2). A maximum likelihood phylogeny constructed with COI sequences (Figure 2) further supports our suspicions that the *“D. nebulosa”* misidentified line is *D. sucinea* and that “*D. sucinea”* 14030–0791.00 is not a *D. sucinea* line. The consistency between our data, data sequenced by others (Khallaf et al., 2021) and uploaded to NCBI, and the freshly obtained lines indicates “*D. nebulosa”* 14030–0761.01 and “*D. sucinea”* 14030–0791.00 strains are misidentified at the NDSSC. We have notified the NDSSC and recommend these strains be used with caution.

Other than revised table and figure text to correct the species misidentification, this issue does not affect any of the results presented by this work.

References to *D. nebulosa* are now revised to D. sucinea** in Figures 1, 2, 3, and 5. Figures legends and the underlying data have not changed.

The corrected Figure 1 is shown here:

**Figure:**
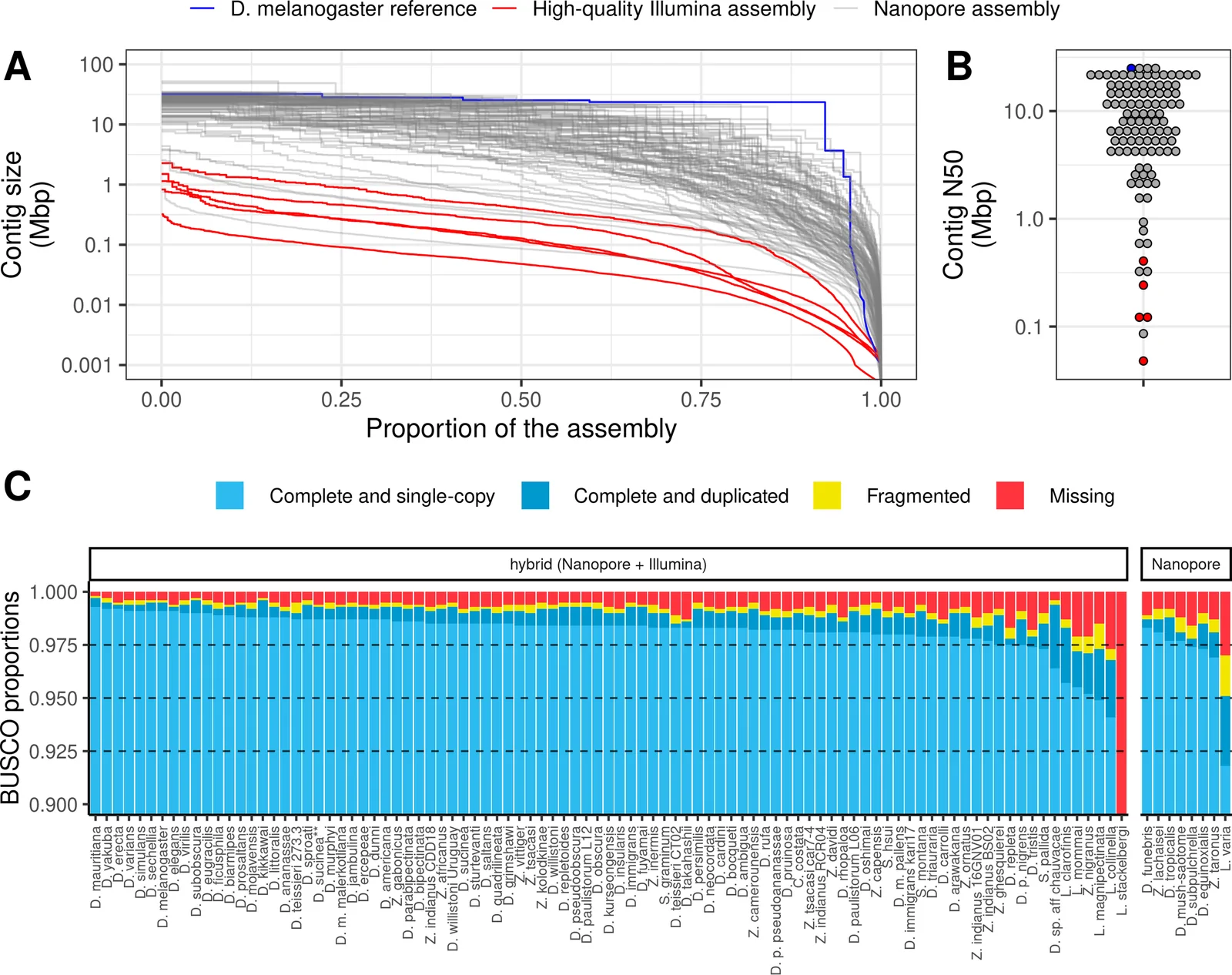


For reference, the originally published Figure 1 is shown:

**Figure:**
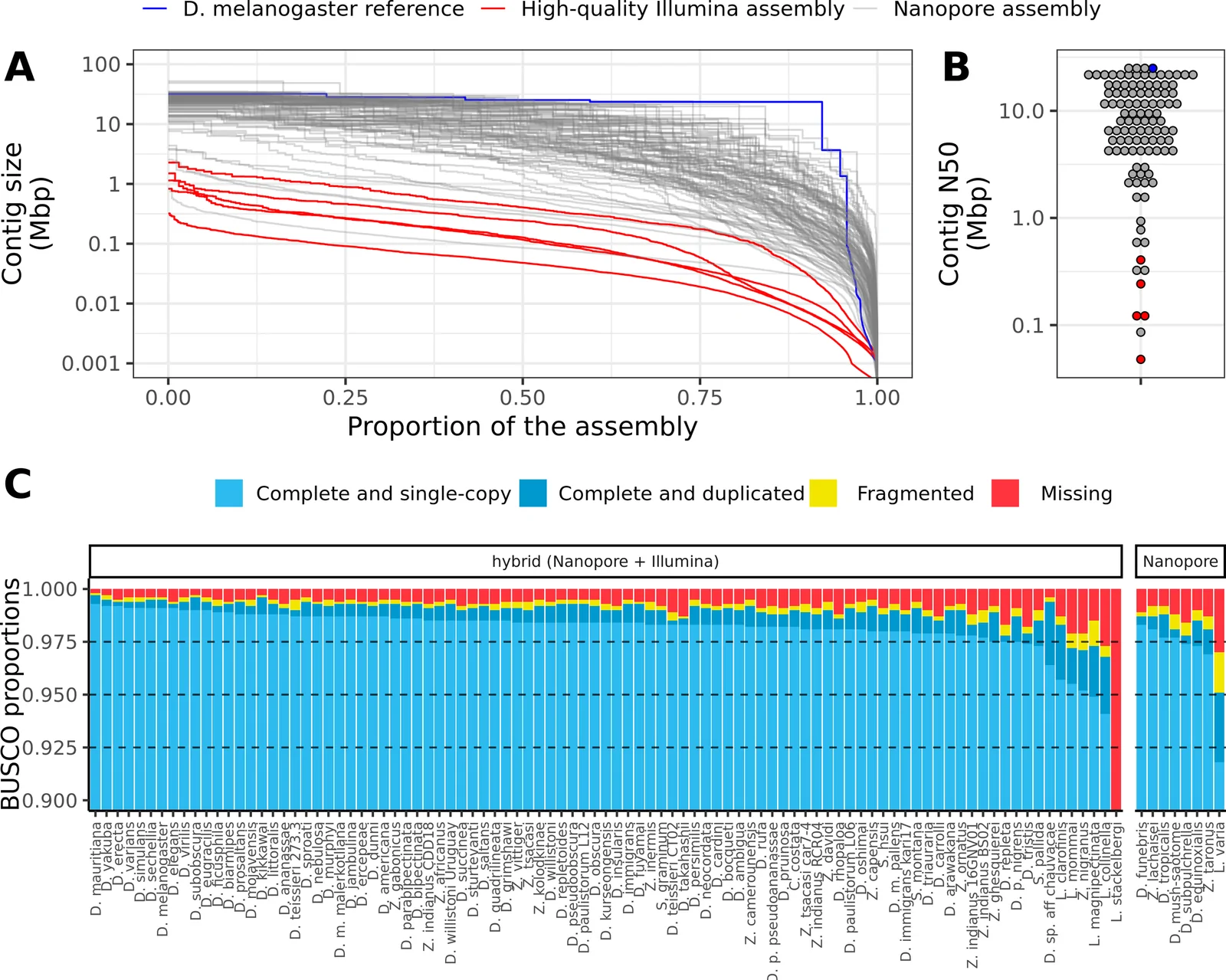


The corrected Figure 2 is shown here:

**Figure:**
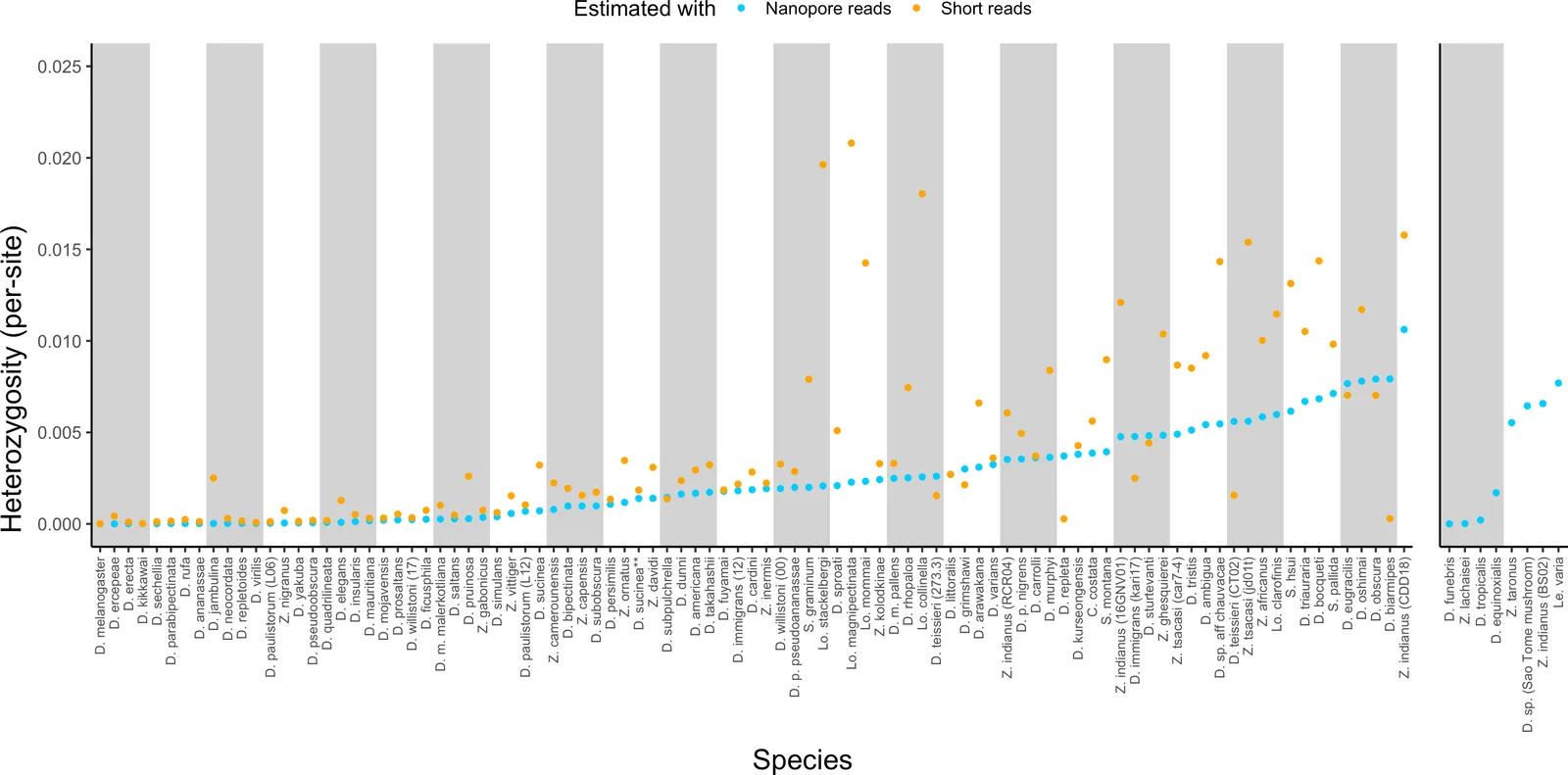


For reference, the originally published Figure 2 is shown:

**Figure:**
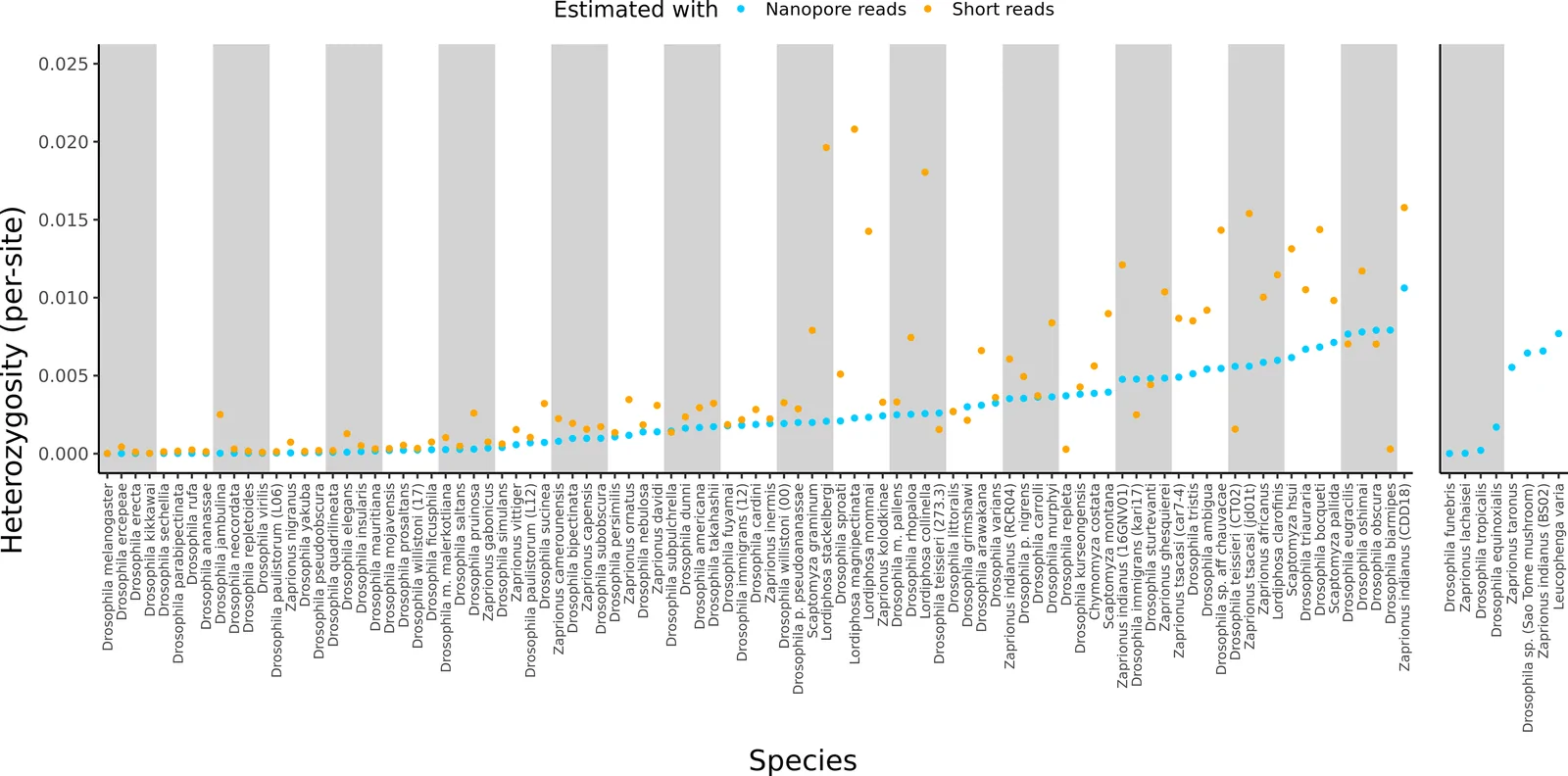


The corrected Figure 3 is shown here:

**Figure:**
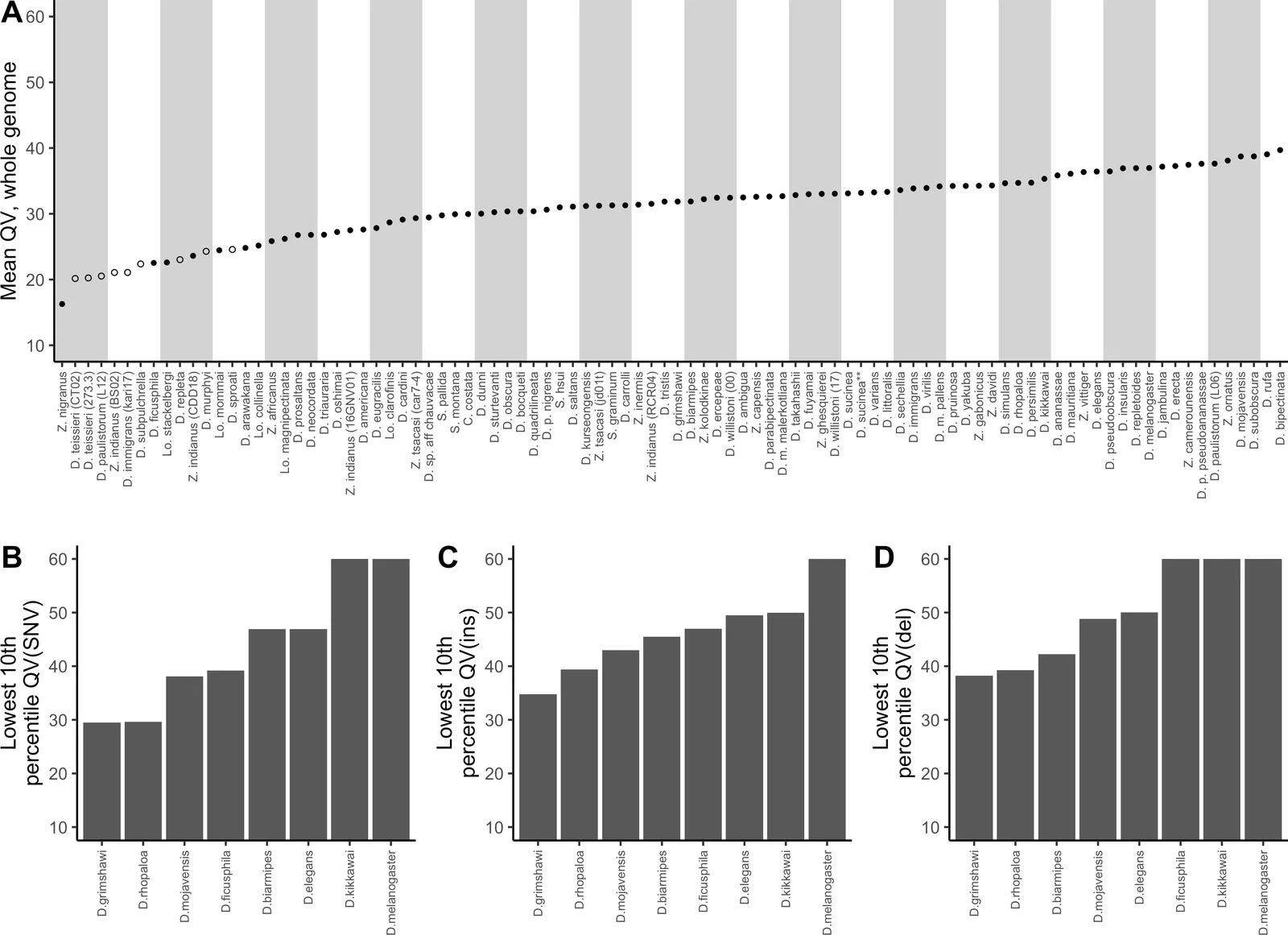


For reference, the originally published Figure 3 is shown:

**Figure:**
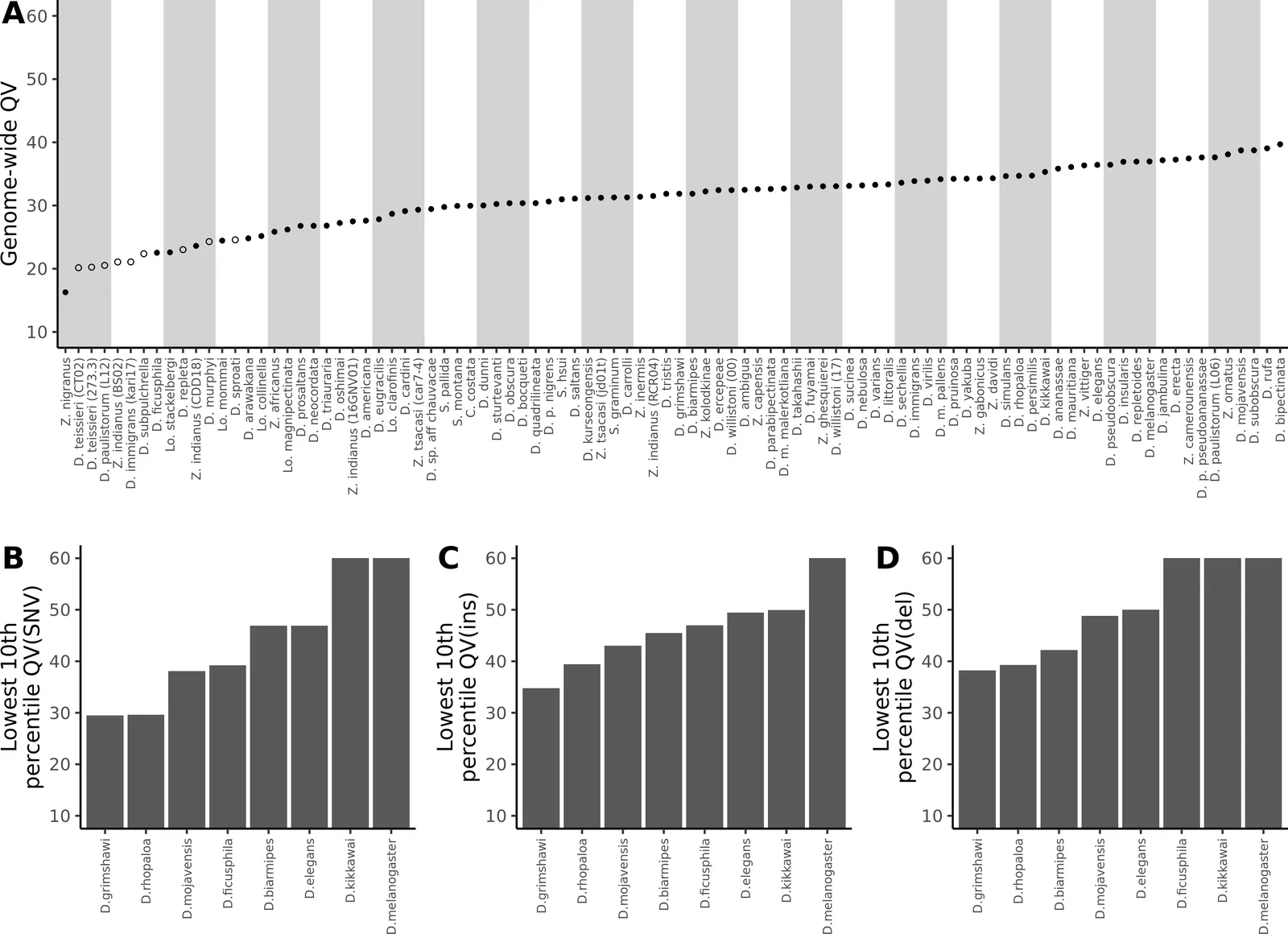


The corrected Figure 5 is shown here:

**Figure:**
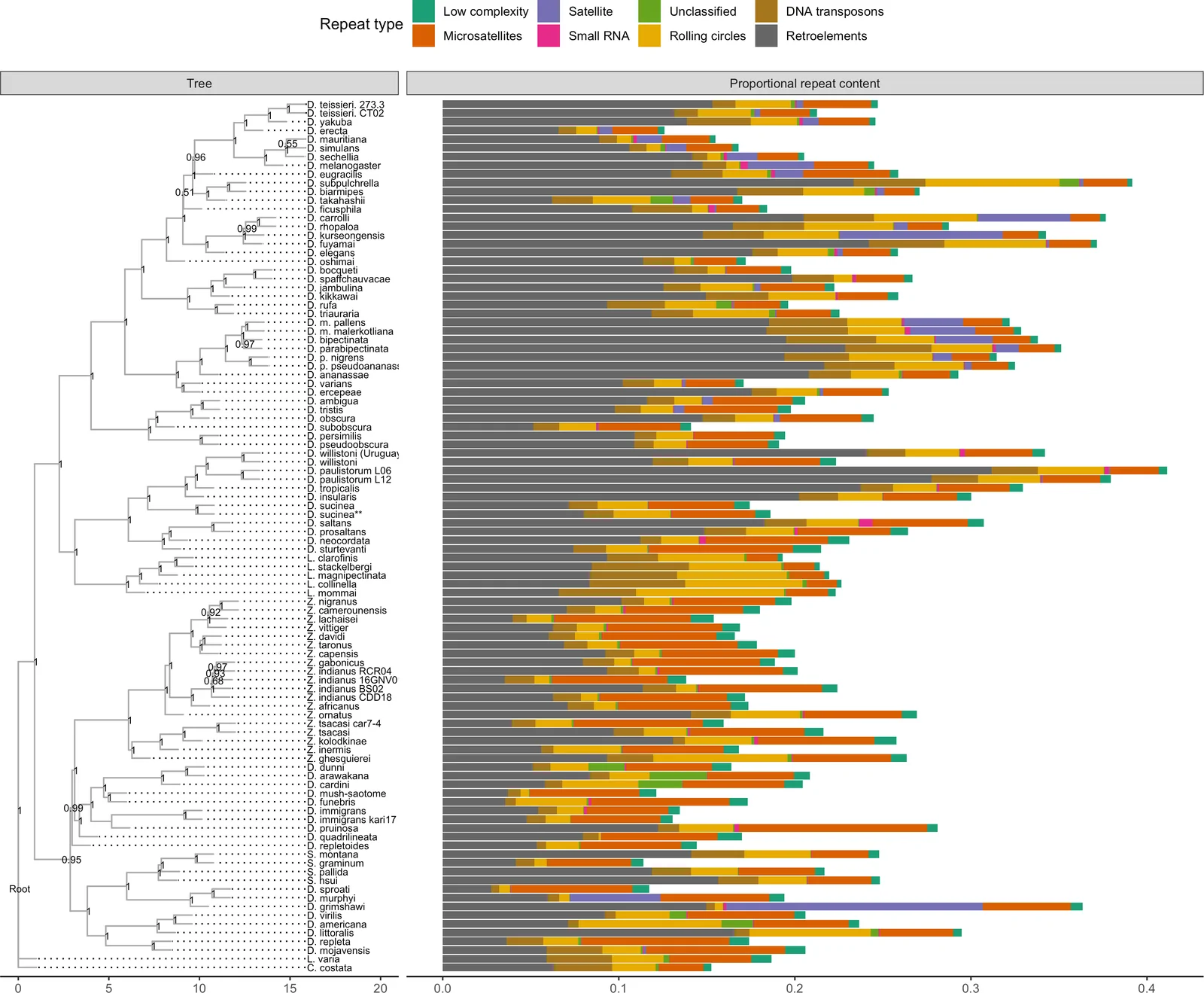


For reference, the originally published Figure 5 is shown:

**Figure:**
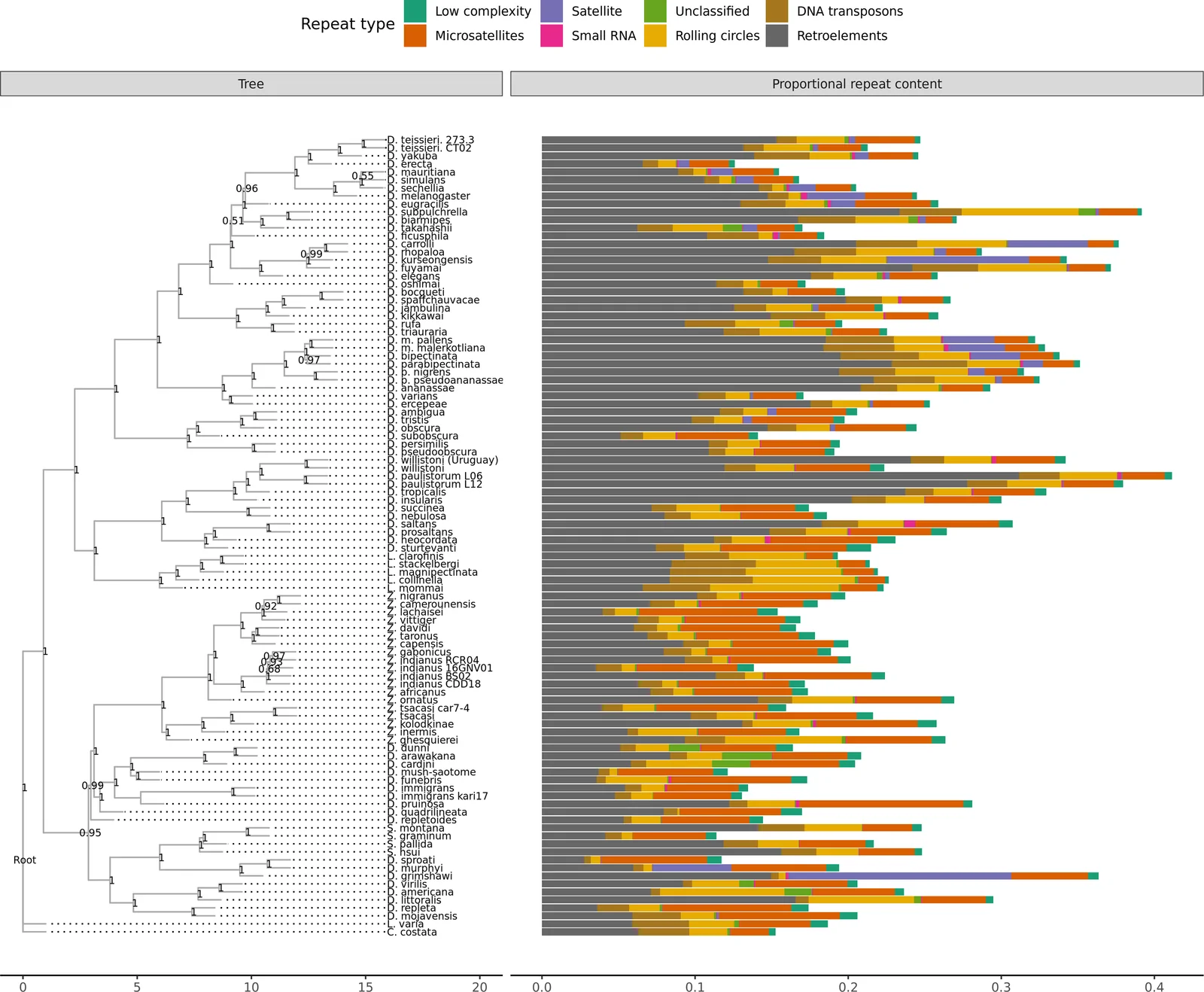


Lastly, any references to *D. nebulosa* in Supplementary Files 1, 2, 3, 4, and 6, and Table 1, are now revised to D. sucinea**. No other entries in these tables are changed.

The article has been corrected accordingly.

**Data accessibility:** Wing photographs are available on Dryad (https://datadryad.org/stash/share/n7sjF2ckUQS2kTcEJJPPMyD4QOFoY0DbWQH9vRrXpfM). NCBI Accession numbers for new COI sequences are listed in Table 1.

**Table 1.** GenBank accession numbers for new COI sequences.

Species	NDSSC Stock #	GenBank accession
*Drosophila capricorni*	14030–0721.01	OK393688
*Drosophila nebulosa*	14030–0761.00	OK393689
*“Drosophila nebulosa”*	14030–0761.01	OK393690
*Drosophila nebulosa*	14030–0761.03	OK393691
*Drosophila nebulosa*	14030–0761.06	OK393692
*“Drosophila sucinea”*	14030–0791.00	OK393693
*Drosophila sucinea*	14030–0791.01	OK393694
*Drosophila sucinea*	14030–0791.02	OK393695
*Drosophila willistoni*	14030–0811.17	OK393696

## References

[bib1] Finet C, Kassner VA, Carvalho AB, Chung H, Day JP, Day S, Delaney EK, De Ré FC, Dufour HD, Dupim E, Izumitani HF, Gautério TB, Justen J, Katoh T, Kopp A, Koshikawa S, Longdon B, Loreto EL, Nunes MDS, Raja KKB, Rebeiz M, Ritchie MG, Saakyan G, Sneddon T, Teramoto M, Tyukmaeva V, Vanderlinde T, Wey EE, Werner T, Williams TM, Robe LJ, Toda MJ, Marlétaz F (2021). DrosoPhyla: resources for drosophilid phylogeny and systematics. Genome Biology and Evolution.

[bib2] Khallaf MA, Cui R, Weißflog J, Erdogmus M, Svatoš A, Dweck HKM, Valenzano DR, Hansson BS, Knaden M (2021). Large-scale characterization of sex pheromone communication systems in *Drosophila*. Nature Communications.

[bib3] Kolmogorov M, Yuan J, Lin Y, Pevzner PA (2019). Assembly of long, error-prone reads using repeat graphs. Nature Biotechnology.

[bib4] Manni M, Berkeley MR, Seppey M, Simão FA, Zdobnov EM (2021). BUSCO update: novel and streamlined workflows along with broader and deeper phylogenetic coverage for scoring of eukaryotic, prokaryotic, and viral genomes. Molecular Biology and Evolution.

[bib5] Prjibelski A, Antipov D, Meleshko D, Lapidus A, Korobeynikov A (2020). Using SPAdes de novo assembler. Current Protocols in Bioinformatics.

[bib6] Ratnasingham S, Hebert PDN (2007). BOLD: The Barcode of Life Data System. Molecular Ecology Notes.

[bib7] Zhang C, Rabiee M, Sayyari E, Mirarab S (2018). ASTRAL-III: polynomial time species tree reconstruction from partially resolved gene trees. BMC Bioinformatics.

